# Effect of intercropping with legumes at different rates on the yield and soil physicochemical properties of *Cyperus esculentus* L. in arid land

**DOI:** 10.3389/fpls.2024.1351843

**Published:** 2024-02-28

**Authors:** Xin Shen, Yalan Liu, Xiangyi Li, Lei Li

**Affiliations:** ^1^ State Key Laboratory of Desert and Oasis Ecology, Xinjiang Institute of Ecology and Geography, Chinese Academy of Sciences, Urumqi, China; ^2^ Xinjiang Key Laboratory of Desert Plant Roots Ecology and Vegetation Restoration, Xinjiang Institute of Ecology and Geography, Chinese Academy of Sciences, Urumqi, China; ^3^ Cele National Station of Observation and Research for Desert-Grassland Ecosystems, Cele, China; ^4^ University of Chinese Academy of Sciences, Beijing, China

**Keywords:** *Cyperus esculentus* L., legumes, intercropping, biomass, plant nutrients, soil properties

## Abstract

Intercropping has the potential to enhance yields and nutrient availability in resource-limited agricultural systems. However, the effects on crop yield nutrients and soil properties can vary considerably depending on the specific plant combinations and intercropping ratios used. In this study, the advantages and impacts of intercropping *C. esculentus* with legumes were investigated by measuring their biomass, nutrient content, and soil properties. The experiment included five intercropping treatments: monoculture of *C. esculentus* (MC), intercropping of *C. esculentus* with *Medicago sativa* L. (alfalfa) at row spacing ratios of 4:4 (4:4CM) and 8:4 (8:4CM), and intercropping of *C. esculentus* with *Glycine max* (L.) Merr. (soybean), also at row spacing ratios of 4:4 (4:4CG) and 8:4 (8:4CG). Our results demonstrated that all four intercropping treatments (4:4CM, 4:4CG, 8:4CM, and 8:4CG) significantly increased the biomass of *C. esculentus* by approximately 41.05%, 41.73%, 16.08%, and 18.43%, respectively, compared with monoculture cultivation alone, among which the 4:4CG treatment was optimum. However, no significant differences were observed in alfalfa or soybean biomass across different intercropping ratios. A notable increase was found in the total nitrogen (TN) and total phosphorus (TP) contents in the leaves, roots, and tubers of *C. esculentus* under intercropping, along with increased soil organic carbon (SOC), alkaline-hydrolyzed nitrogen (AN), available phosphorus (AP), microbial biomass carbon (MBC), microbial biomass nitrogen (MBN), and soil water content (SWC), and significantly reduced the soil pH. Among the intercropping treatments, the 4:4CG treatment also exhibited the most favorable soil properties. In particular, compared with MC, the 4:4CG treatment resulted in significant increases of 163.8%, 394.6%, and 716.8% in SOC, AN, and AP contents, respectively. The same treatment also led to significant increases of 48.34%, 46.40%, and 208.65% in MBC, MBN, and SWC, respectively. Overall, the findings suggest that the use of 4:4CG intercropping is an effective approach for sustainable farming management in Xinjiang.

## 1 Introduction

Approximately 45% of the Earth’s land area comprises drylands (Prăvălie, 2016), and with the ongoing climate warming, these global drylands are projected to expand in the forthcoming decades (Zhang et al., 2021). However, such an expansion poses a remarkable threat to food security, thus underscoring the urgent need to identify suitable crops and cultivation methods for these dry regions. *C. esculentus*, commonly known as tiger nut and belonging to the family *Cyperaceae Juss.*, is indigenous to the Mediterranean region of Africa (Follak et al., 2016). It is widely distributed in many northern temperate regions and is becoming increasingly popular as an energy crop in China, India, Egypt, and the United States (Ayeni, 2022). *C. esculentus* possesses exceptional traits, such as drought resistance and salinity tolerance (Duan et al., 2022; Zhang et al., 2023; Zhu et al., 2023). The tubers of *C. esculentus* are rich in fats, proteins, sugars, other nutrients, and a wide range of active compounds, making it a high-quality, high-yield, and high-value crop (Coskuner et al., 2002). In addition, the above-ground leafy part of *C. esculentus*, which has a growing period of around 120 days, is usually harvested for animal fodder, while the underground tuber part can be used as high-quality fodder, making it an ecologically vital cash crop (Kizzie-Hayford et al., 2015).

Intercropping, which refers to the concurrent cultivation of two or more crops on the same plot (Willey, 1990), offers heightened crop yield and stability compared with monocropping (Lan et al., 2023; Parvin et al., 2023). This agricultural practice mitigates the adverse environmental impacts associated with modern farming methods while simultaneously optimizing soil nutrient, water, and resource utilization (Chen et al., 2017). Various common species can be utilized for intercropping (Qu et al., 2023), and studies have demonstrated notable advantages when legumes are included—for instance, Qu et al. (2022) revealed that rape with common vetch significantly increased biomass, reduced soil pH, and increased soil organic carbon (SOC) and soil available phosphorus (AP). Similarly, Huang et al. (2022) demonstrated that the intercropping of tea plant with both soybean and zoysia significantly increased SOC, total nitrogen (TN), and tea quality. These findings can be attributed to the ability of legumes to alleviate nutrient stress and enhance nutrient availability through nitrogen (N) fixation or transfer to non-leguminous plants (Hobbie and Högberg, 2012; Shao et al., 2020). In addition, legumes serve as nutrient suppliers by delivering nutrients to non-leguminous plants via root secretions or arbuscular mycorrhizal fungal networks (Hauggaard-Nielsen and Jensen, 2005; Li et al., 2008; Adomako et al., 2022). Together with the root exudates, the legume-based intercropping system also promotes a beneficial rhizobacterial community, thereby improving soil quality and the rhizosphere soil environment for better resource uptake (Duchene et al., 2017; Chamkhi et al., 2022; Zhao et al., 2022). This synergistic effect on nutrient acquisition contributes to the growth of both intercropped species, ultimately leading to increased crop yield (Lu et al., 2024).

In addition, Black and Ong (2000) reported that, compared with monocropping, intercropping reduced crop water consumption (WC) without affecting total biomass production. Rezig et al. (2013) also showed that intercropping led to WC reduction in potato and sulla, resulting in increased water use efficiency of both crops. This phenomenon can be attributed to two factors. First, intercropping systems can improve water use efficiency and land occupancy by significantly developing canopy and reducing soil evapotranspiration. Second, the proportion of evapotranspiration flow is higher in intercropping than in monoculture, resulting in an expansion of the vegetation cover area and a subsequent increase in evapotranspiration. In turn, this reduces soil evapotranspiration in intercropping systems and increases soil water content (SWC). Therefore, it is important to study different intercropping treatments to increase crop yield and plant nutrients, improve soil nutrients, reduce soil pH, and increase SWC in arid areas.

In intercropping systems, the growth and resource capture of crops are determined by both intraspecific and interspecific competition among plants (Vandermeer, 1989). Therefore, maintaining appropriate ratios in intercropping systems is crucial to mitigate plant competition and optimize environmental resource uptake (Koocheki et al., 2016). Typically, a higher proportion of legumes can enhance atmospheric N fixation through symbiotic interactions with rhizobia, thus mitigating competition with non-leguminous plants for soil N resources; however, increasing proportions of intercropped plants may lead to reduced yields from the main crop (Andersen et al., 2005; Viaud et al., 2023). In addition, legumes possess the ability to enhance N availability in intercropping systems by fixing N, minimizing N leaching, and supplying N to neighboring crops (Vrignon-Brenas et al., 2016), yet whether this increased N availability offsets the competitive effects of higher proportions remains unclear from existing findings. Therefore, different intercropping proportions were also explored in the current study to investigate whether increased N availability from intercropping with legumes could counteract the competitive nature of high proportions of *C. esculentus* and whether it could improve *C. esculentus* yields and nutrients.

The southern fringe of the Taklamakan Desert in Xinjiang is located within the arid and semiarid belt of Asia and Europe, which features a typical temperate continental arid climate (Mazdiyasni and AghaKouchak, 2015). Xinjiang’s predominant sandy soil, coupled with the snakeberry’s robust root system and vigorous tillering capacity, contributes to sand fixation, soil stabilization, and wind protection (Aydar et al., 2020). *Medicago sativa* L. (alfalfa) and *Glycine max* (L.) Merr. (soybean) are both major legume crops in this region, which can significantly enhance the fertility and physicochemical properties of soil, thus establishing a mutually beneficial relationship between utilization and conservation (Rau et al., 2023). Alfalfa and soybean are sensitive to drought, with limited growth and lower yields in dry areas. However, some studies have demonstrated that intercropping with other crops can reduce the drought sensitivity of alfalfa and soybean, reduce water consumption, and increase yields (Ouda et al., 2007; Mao et al., 2012; Zhang et al., 2021; Jamali et al., 2023). In view of the limited research on intercropping *C. esculentus*, an emerging oilseed crop, the present study presents an innovative approach to intercropping *C. esculentus* with alfalfa and soybean in different proportions to assess the effects on their yield, nutrients, and soil properties. The goal of the current work is to fully utilize the economic and ecological potential of *C. esculentus* and to understand the key methods of growing oil salsa bean in such drylands. Furthermore, this study aims to determine whether intercropping two legumes can alleviate the competitive effects caused by the high rates of *C. esculentus*. To this end, two hypotheses were formulated: (1) intercropping significantly enhances the yield and nutrients of *C. esculentus* and enriches soil nutrient content and (2) legumes effectively counteract the competitive effects among *C. esculentus* when intercropped with high proportions of *C. esculentus*.

## 2 Material and methods

### 2.1 Site description

**Figure 1:**
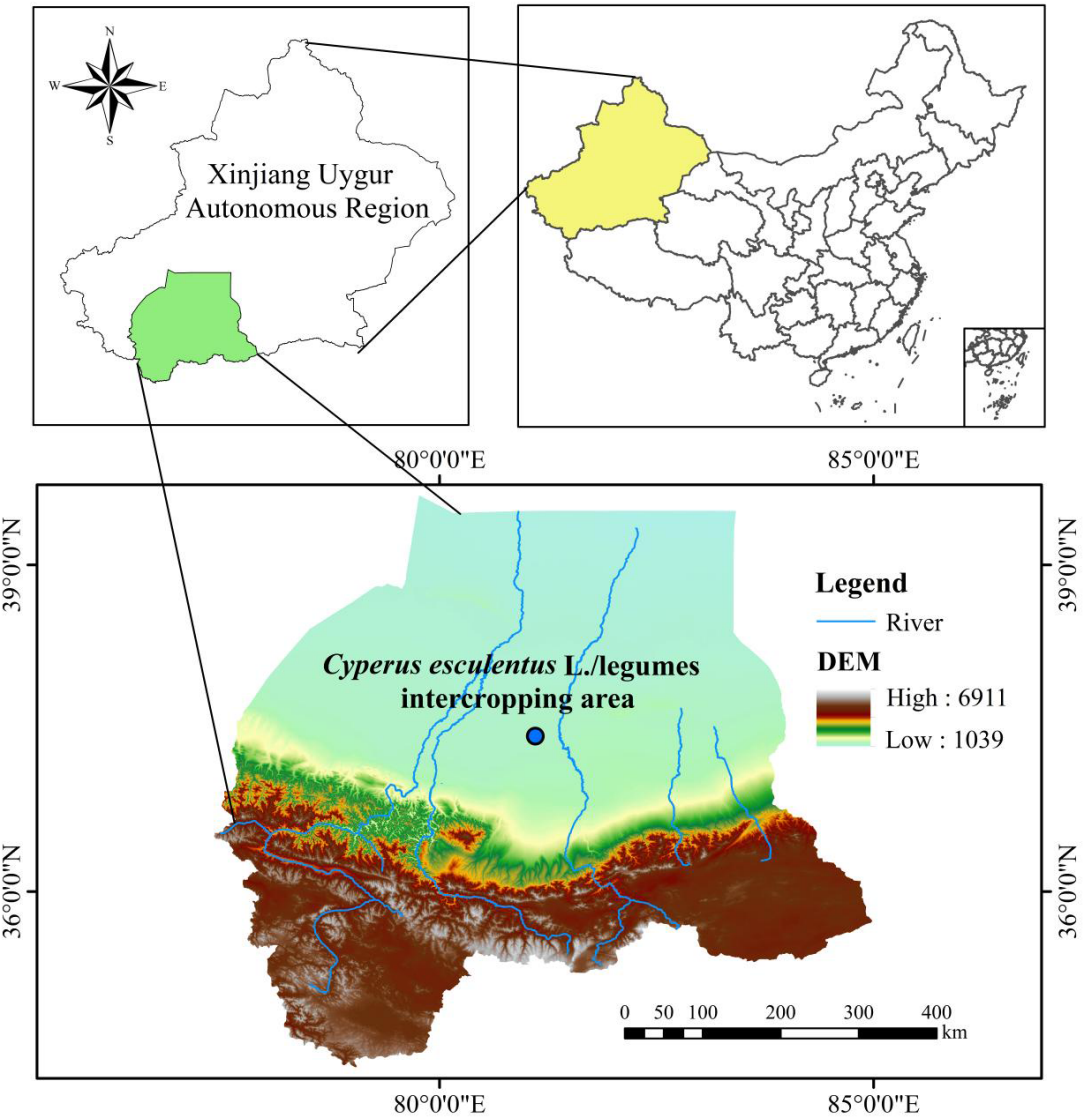
Location of the *C. esculentus* planting demonstration area in Hotan Prefecture, Xinjiang, China.

This experiment was conducted in June 2021 in the *C. esculentus* planting area (81°06′18″ E, 37°25′99″ N) located in Tuan Jie New Village in the Hotan Prefecture of the Xinjiang Uygur Autonomous Region, China (**Figure 1**). The area is a newly reclaimed sandy land located in the Taklamakan Desert that features a temperate continental desert climate, with an average annual temperature of approximately 13.1°C, an average annual precipitation of 43.8 mm, and a total evapotranspiration of 2,624.4 mm. The initial soil indicators were collected from the area at depths of 0–20 cm, air-dried, and sieved through a 2-mm sieve to remove rhizomes and debris. Next, the physical and chemical properties of the soils were determined with three replicates for all measurements, thereby yielding the following results: soil capacity (1.54 g·cm^−3^), pH (8.72), SOC (2.11 g·kg^−1^), AN (1.86 mg·kg^−1^), AP (1.23 mg·kg^−1^), MBC (23.09 mg·kg^−1^), and MBN (2.16 mg·kg^−1^).

### 2.2 Experimental design

**Figure 2:**
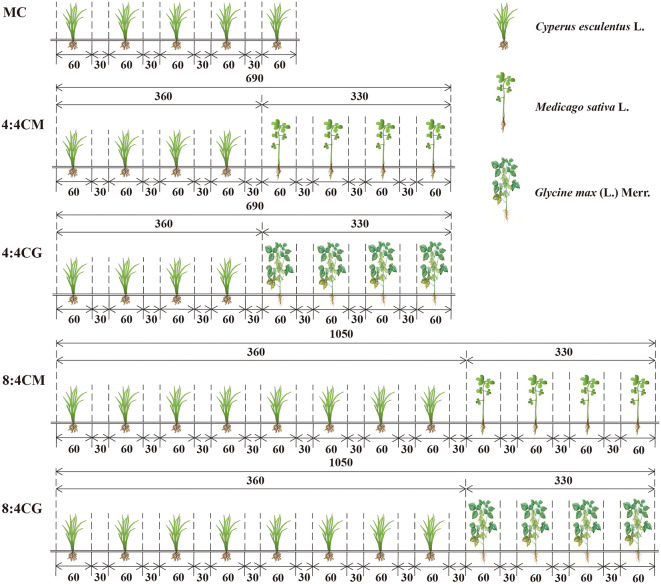
Diagram of different intercropping treatments of *C. esculentus* with alfalfa and soybean (the numbers indicate the distance in centimeters). MC, 4:4CM, 4:4CG, 8:4CM, and 8:4CG represent monocropping *C. esculentus*, 4:4 *C. esculentus*/Medicago sativa L. intercropping, 4:4 *C. esculentus*/*Glycine max* (L.) Merr., 8:4 *C. esculentus*/*Medicago sativa* L., and 8:4 *C. esculentus* L./*Glycine max* (L.) Merr. intercropping, respectively.

The experimental variety of *C. esculentus* used in this study was “XinKe No. 1.” The experiment followed a one-way randomized design, including the following five treatments: *C. esculentus* monoculture (MC), *C. esculentus* and alfalfa according to a 4:4 intercropping (4:4CM), *C. esculentus* and soybean according to a 4:4 intercropping (4:4CG), *C. esculentus* and alfalfa according to an 8:4 intercropping (8:4CM), and *C. esculentus* and soybean according to an 8:4 intercropping (8:4CG). All rows were uniformly spaced at intervals of 30 cm, and each row strip measured 60 cm in width (**Figure 2**). Each treatment plant was planted in a one-hectare experimental plot. A base fertilizer comprising 300 kg·hm^−2^ urea (*N* ≥ 18%), 300 kg·hm^−2^ diammonium phosphate (P_2_O ≥ 48%), and 300 kg·hm^−2^ humic acid was applied, while water application amounted to 2,025 m^3^·hm^-2^.

The sowing process took place on June 10, 2021 using mechanically opened furrows and strip planting techniques. The sowing rate for the planting treatments remained constant at 300 kg·hm^−2^. Throughout the growing season, fertilizers and water were administered via drip irrigation, with each drip irrigation strip spaced at intervals of 30 cm apart. In the early growth stage of *C. esculentus* (July), water was dripped four times, with a total volume of approximately 9.38 m^3^·hm^-2^, along with an application of approximately 1.69 m^3^·hm^-2^ urea and 0.75 kg·hm^-2^ potassium sulfate (*K* ≥ 52%). During the middle stage of irrigation (from August to the end of September), water was applied 12 times, followed by additional four applications during the late stage of irrigation (from the end of September to the beginning of October), using identical quantities for both water and fertilizer inputs. Using appropriate herbicides and pesticides, weed and pest control measures were implemented at regular monthly intervals.

The resulting plants were harvested on October 10, 2021. Three 10 × 5-m plots were set up at 5 m apart. Three designated sampling points were selected in each plot, and plant samples (leaves, roots, and tubers) were collected from the central areas (0.5 m × 0.5 m) of these points, with 10 plants randomly selected from each point to record above- and below-ground biomass. Next, soil samples were collected from three random points in each plot using a soil sampler; each point had a 2-cm diameter and a depth range of 0–20 cm. All soil samples were thoroughly mixed to form a uniform sample and then passed through a 2-mm sieve to remove roots and debris.

### 2.3 Sampling and measurements

The plant samples were washed with water, purified, and dried at 75°C for 48 h until the biomass was constant and then subjected to the determination of indicators. Subsequently, the dry matter was ground using a vibrating disc mill (RS200, Retsch GmbH Inc., Haan, Germany). After passing through a 1-mm sieve, the nutrient content and quality of the different organs were assessed. Organic carbon (OC) and total nitrogen (TN) concentrations were measured using a CN autoanalyzer (Eurovector, Milan, Italy), and total phosphorus (TP) concentrations were analyzed by using Mo–Sb colorimetric method after persulphate oxidation (Lu et al., 2015).

The soil samples were passed through a 2-mm sieve to eliminate rhizomes and debris. Subsequently, the samples were air-dried at 105°C for 48 h. The soil pH was determined using a pH meter (PHS-3C, China), while soil organic carbon (SOC) content was assessed using the potassium dichromate oxidation-heating method (Jiang et al., 2017). Soil alkali-hydrolyzable nitrogen (AN) concentration was quantified through the Kjeldahl method (Bremner and Mulvaney, 1982), while AP content was measured using the ammonium molybdate method after completing the H_2_SO_4_ digestion of the samples (Liu et al., 2021). Soil microbial biomass carbon (MBC) and microbial biomass nitrogen (MBN) were quantified via fumigation extraction (Vance et al., 1987). The soil samples were weighed and measured both before and after being air-dried in an oven at 105°C to determine the SWC and soil bulk density (Chehab et al., 2020). The particle size distribution of the soil was analyzed using the pipette method (Chen et al., 1991). Following the guidelines of the International System of Soil Classification (ISSC), the particle sizes were classified into four categories: clay (0–0.002 mm), silt (0.002–0.02 mm), very fine sands (Vfs, 0.02–0.2 mm), and medium and coarse sands (Macs, 0.2–2 mm).

### 2.4 Statistical analysis

The effects of intercropping on the nutrients, above- and below-ground biomass, and soil properties of *C. esculentus* were tested using one-way analysis of variance (ANOVA). Tukey’s test was employed to compare differences among intercropping treatments. All statistical analyses were performed using R v4.1.0 (R Core Team, 2018) and SPSS 23.0 software (SPSS Inc., Chicago, IL, USA), with the significance level set at *P* < 0.05. Pearson correlation analysis with the corrplot software package was utilized to investigate and visualize plant–soil relationships.

## 3 Results

### 3.1 Effect of different intercropping treatments on the biomass of *C. esculentus*

The biomass obtained from the 4:4CM and 4:4CG treatments of oilseed bean was significantly higher (*P* < 0.05) than that obtained via the MC treatment, with increases of 41.05% and 41.73%, respectively (**Table 1**). The biomass of oilseed bean in the 8:4CM and 8:4CG treatments was not significantly different (*P* > 0.05) from that of the MC treatment. Furthermore, the biomass of alfalfa and soybean did not differ significantly (*P* > 0.05) among different intercropping treatments. The *C. esculentus* leaf biomass, produced by the 4:4CM treatment, was significantly higher than that produced by MC at 56.85% (*P* < 0.05), while those produced by the 4:4CG, 8:4CM, and 8:4CG treatments were not significantly different from that produced by the MC treatment (*P* > 0.05) (**Table 2**). Regarding the root biomass of *C. esculentus*, the 4:4CG treatment yielded significantly higher biomass by 78.41% (*P* < 0.05) than MC. In addition, for tuber biomass, both 4:4CM and 4:4CG treatments yielded significantly higher tuber biomass than the MC treatment by 29.77% and 39.01% (*P* < 0.05), respectively, while the 8:4CM and 8:4CG treatments did not have any significant (*P* > 0.05) difference from MC.

**Table 1:** Total biomass of *C. esculentus*, alfalfa, and soybean under different intercropping treatments (kg·acre^-2^/g).

Treatment	Biomass		
	*C. esculentus*	*Medicago sativa* L.	*Glycine max* (L.) Merr.
MC	272.98 ± 28.61 b	–	–
4:4CM	385.04 ± 39.11 a	198.68 ± 41.33 a	–
4:4CG	386.89 ± 58.28 a	–	217.40 ± 35.87 a
8:4CM	316.88 ± 10.18 ab	187.29 ± 54.54 a	–
8:4CG	334.65 ± 42.65 ab	–	200.63 ± 29.22 a

The different lowercase letters indicate significant differences between intercropping treatments (P < 0.05). MC, 4:4CM, 4:4CG, 8:4CM, and 8:4CG represent monocropping *C. esculentus*, 4:4 *C. esculentus/Medicago sativa* L. intercropping, 4:4 C. esculentus/Glycine max (L.) Merr., 8:4 *C. esculentus/Medicago sativa* L., and 8:4 *C. esculentus/Glycine max* (L.) Merr. intercropping, respectively. In addition, there were no data for *Medicago sativa* L. and *Glycine max* (L.) Merr. because MC represents monoculture *C. esculentus*, no data for *Glycine max* (L.) Merr. because 4:4CM and 8:4CM represent an intercropping of *C. esculentus* and *Medicago sativa* L., and no data for *Medicago sativa* L. because 4:4CG and 8:4CG represent an intercropping of *C. esculentus* and *Glycine max* (L.) Merr. The symbol “-” in the table means no data.

**Table 2:** Biomass of the separate components of *C. esculentus* under various intercropping treatments (kg·acre^-2^/g).

Organ	Treatment				
	MC	4:4CM	4:4CG	8:4CM	8:4CG
Leaf	101.60 ± 19.02 b	159.36 ± 32.15 a	137.01 ± 36.61 ab	126.18 ± 26.72 ab	128.44 ± 24.02 ab
Root	29.51 ± 3.48 d	41.56 ± 6.19 bc	52.65 ± 5.38 a	33.79 ± 3.33 cd	43.51 ± 3.51 b
Tuber	141.87 ± 7.81 c	184.11 ± 16.98 ab	197.22 ± 27.09 a	156.91 ± 27.32 bc	162.70 ± 17.17 abc

The different lowercase letters indicate significant differences between intercropping treatments at P < 0.05. MC, 4:4CM, 4:4CG, 8:4CM, and 8:4CG represent monocropping *C. esculentus*, 4:4 *C. esculentus/Medicago sativa* L. intercropping, 4:4 *C. esculentus/Glycine max* (L.) Merr., 8:4 *C. esculentus/Medicago sativa* L., and 8:4 *C. esculentus/Glycine max* (L.) Merr. intercropping, respectively.

### 3.2 Effects of different intercropping treatments on the nutrient concentrations of *C. esculentus*

**Figure 3:**
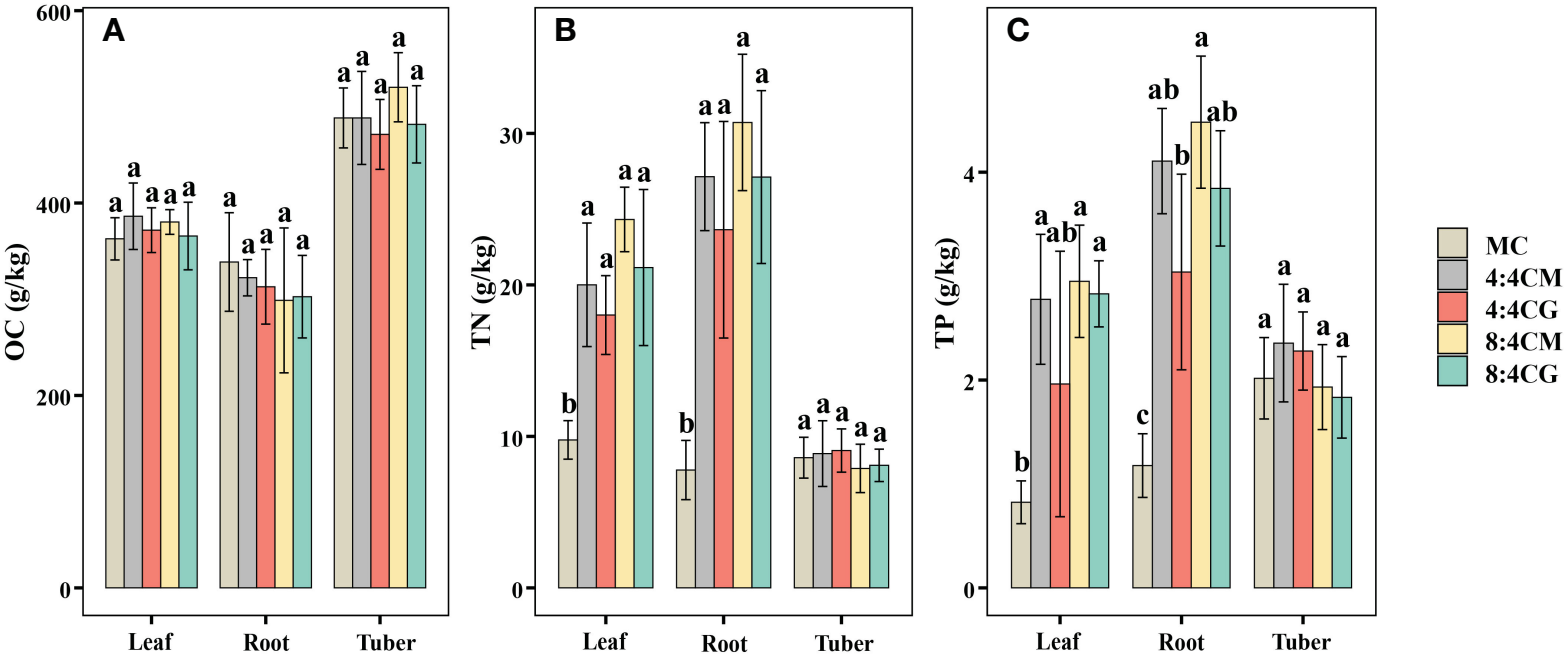
Effect of different intercropping treatments on **(A)** organic carbon content, **(B)** total nitrogen content, and **(C)** total phosphorus content of the leaves, roots, and tubers of *C. esculentus*. The different small letters indicate significant differences among the four treatments (*P* < 0.05). MC, 4:4CM, 4:4CG, 8:4CM, and 8:4CG represent monocropping *C. esculentus*, 4:4 *C. esculentus*/*Medicago sativa* L. intercropping, 4:4 *C. esculentus*/*Glycine max* (L.) Merr., 8:4 *C. esculentus*/*Medicago sativa* L., and 8:4 *C. esculentus*/*Glycine max* (L.) Merr. intercropping, respectively.

The OC contents in leaves, roots, and tubers of *C. esculentus* were not significantly different (*P* > 0.05) in all treatments (**Figure 3A**). In comparison, the TN contents in the leaves and roots of *C. esculentus* were significantly higher (*P* < 0.05) in all the intercropping treatments compared with MC (**Figure 3B**), although there were no significant differences (*P* > 0.05) among the intercropping treatments. The highest TN contents were found in the leaves and roots of aubergine under the 8:4CM treatment, which increased by 149.08% and 295.03%, respectively, compared with the MC treatment. The TP content of *C. esculentus* leaves and roots was significantly higher in the 8:4CM treatment than in MC (**Figure 3C**), with increases of 258.3% and 258.3%, respectively. However, there were no significant differences (*P* > 0.05) among the various intercropping treatments. Furthermore, the TN and TP contents of *C. esculentus* tubers did not show significant differences (*P* > 0.05) among all treatments.

### 3.3 Effect of different intercropping treatments on the soil properties of *C. esculentus*

**Figure 4:**
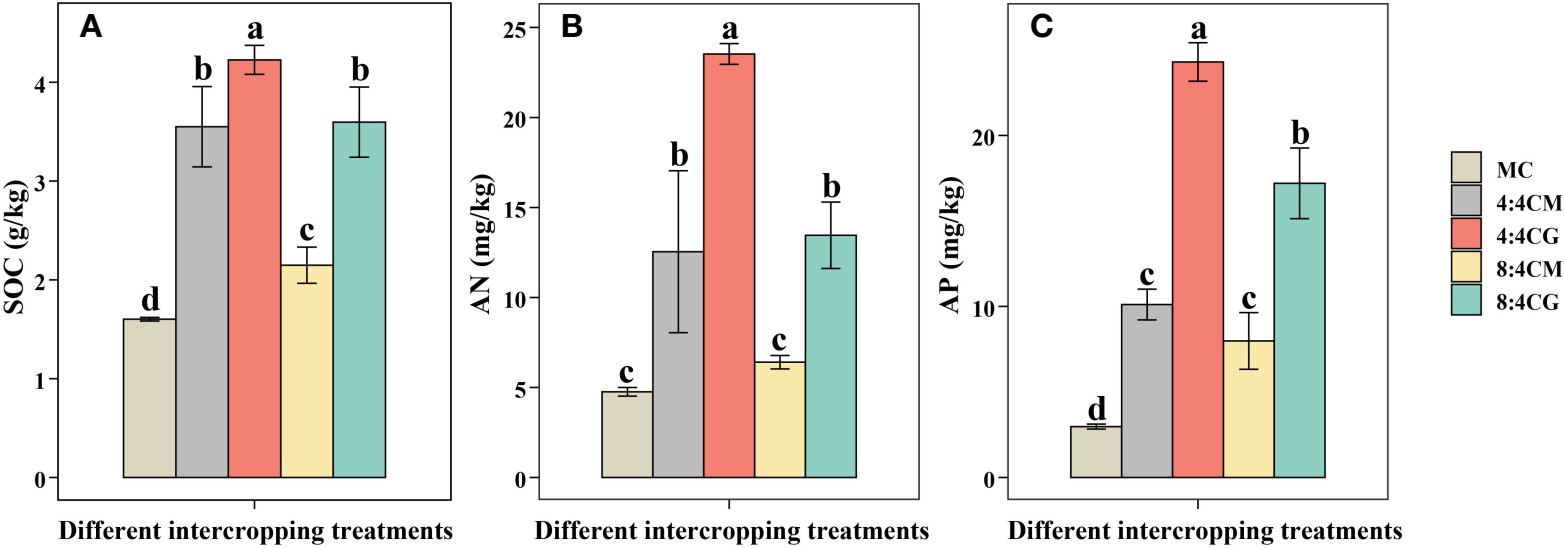
Effects of different intercropping treatments on **(A)** soil organic carbon content, **(B)** soil alkali-hydrolyzable nitrogen content, and **(C)** soil available phosphorus content. The different small letters indicate significant differences among the five treatments (*P* < 0.05). MC, 4:4CM, 4:4CG, 8:4CM, and 8:4CG represent monocropping *C. esculentus*, 4:4 *C. esculentus*/*Medicago sativa* L. intercropping, 4:4 *C. esculentus*/*Glycine max* (L.) Merr., 8:4 *C. esculentus*/*Medicago sativa* L., and 8:4 *C. esculentus*/*Glycine max* (L.) Merr. intercropping, respectively.

All intercropping treatments showed a significant increase in the SOC, with the 4:4CG treatment being the most significant (*P* < 0.05) (**Figure 4A**). In particular, the 4:4CG treatment increased the SOC by 163.89% compared with the MC treatment. In terms of the AN content, the 4:4CM, 4:4CG, and 8:4CG treatments showed significant (*P* < 0.05) increases compared with MC (**Figure 4B**), with the 4:4CG treatment showing the most significant increase of 163.89% compared with the MC. Meanwhile, there was no significant difference (*P* > 0.05) between the 8:4CM treatment and MC. For the AP, all intercropping treatments showed significant (*P* < 0.05) increases compared with MC (**Figure 4C**), with the 4:4CG treatment being the most significant, increasing the AP by 163.89% compared with the MC treatment.

**Figure 5:**
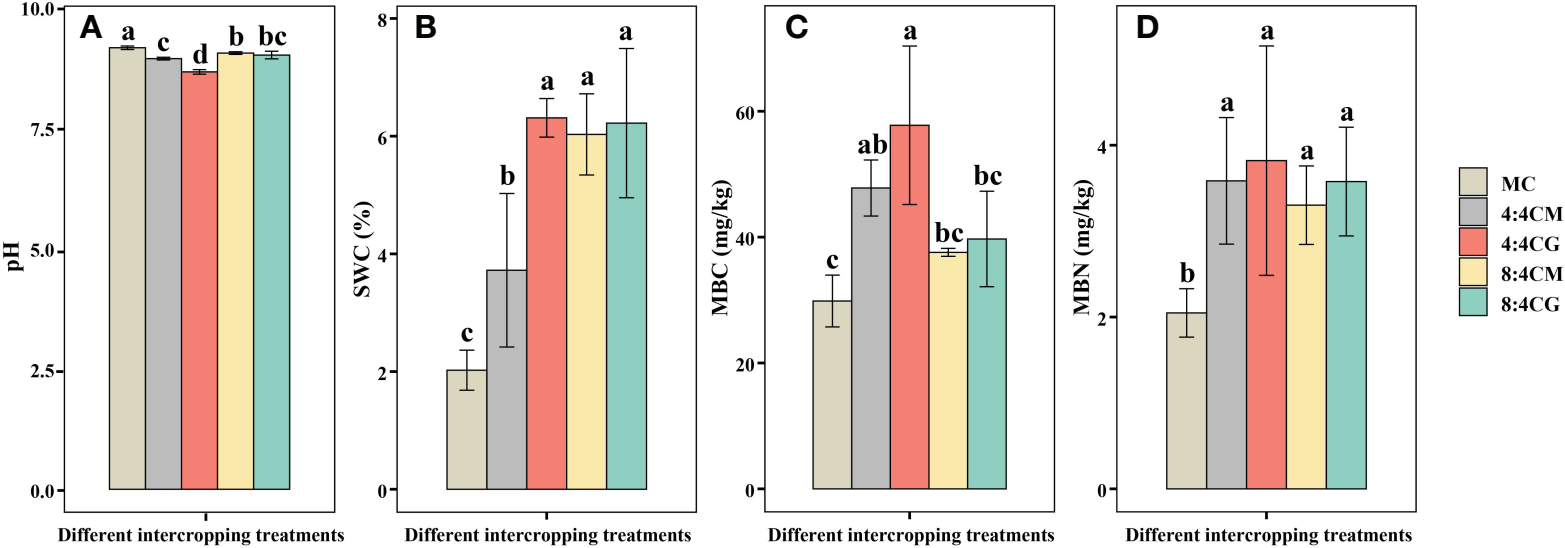
Effects of different intercropping treatments on **(A)** soil pH, **(B)** soil water content (SWC), **(C)** soil microbiomass carbon, and **(D)** soil microbiomass nitrogen content. The different small letters indicate significant differences among the four treatments (*P* < 0.05). MC, 4:4CM, 4:4CG, 8:4CM, and 8:4CG represent monocropping *C. esculentus*, 4:4 *C. esculentus*/*Medicago sativa* L. intercropping, 4:4 *C. esculentus*/*Glycine max* (L.) Merr., 8:4 *C. esculentus*/*Medicago sativa* L., and 8:4 *C. esculentus*/*Glycine max* (L.) Merr. intercropping, respectively.

The soil pH values of all four intercropping patterns were significantly lower than those of the monocropping treatments (**Figure 5A**), with the lowest pH value in the 4:4CG treatment (*P* < 0.05), which was reduced by 5.78%, 3.18%, 4.51%, and 4.01% compared with the MC, 4:4CM, 8:4CM, and 8:4CG treatments, respectively. In addition, SWC was significantly higher (*P* < 0.05) in the intercropping treatments than in the monocropping treatments, with 4:4CG averaging the highest with an increase of 212.07% compared with the MC treatment (**Figure 5B**). MBC was significantly higher (*P* < 0.05) in both the 4:4CM and 4:4CG treatments than MC (**Figure 5C**), with the former being the highest on average, showing an increase of 93.57% compared with MC. However, there was no significant difference between the 8:4CM and 8:4CG intercrop treatments and MC (*P* > 0.05). Moreover, MBN was significantly higher (*P* < 0.05) in the 4:4CM, 4:4CG, 8:4CM, and 8:4CG treatments than in the MC treatment (**Figure 5D**), with increases of 75.09%, 86.57%, 61.25%, and 74.68%, respectively. There were no significant differences (*P* > 0.05) among the intercrop treatments.

**Figure 6:**
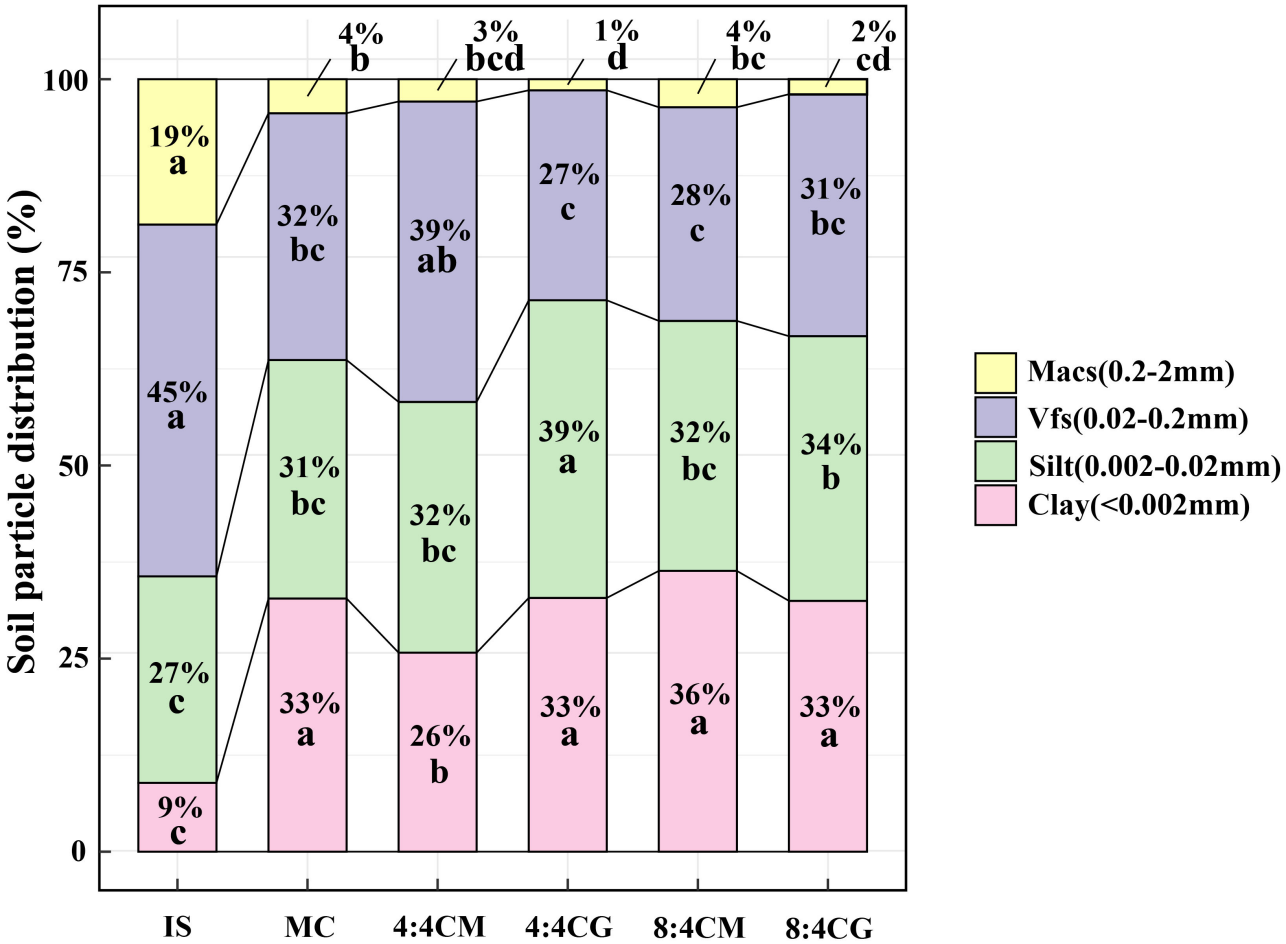
A comparison of original soil samples, monocropping, and intercropping treatments was analyzed to characterize soil particle size distribution. The different small letters indicate significant differences among the four treatments (*P* < 0.05). Vfs and Macs represent very fine sand and medium and coarse sand, respectively. IS, MC, 4:4CM, 4:4CG, 8:4CM, and 8:4CG represent initial soil, monocropping *C. esculentus*, 4:4 *C. esculentus*/*Medicago sativa* L. intercropping, 4:4 *C. esculentus*/*Glycine max* (L.) Merr., 8:4 *C. esculentus*/*Medicago sativa* L., and 8:4 *C. esculentus*/*Glycine max* (L.) Merr. intercropping, respectively.

The MC and intercropping treatments significantly altered the proportion of grain size in the initial soil (IS, **Figure 6**). In the IS, the largest proportion of Vfs was 45%, while the smallest proportion of clay was 9%. However, after the MC and intercropping treatments, the percentage of grain size in the soil changed significantly (*P* < 0.05). There was also a significant decrease in Macs and a significant increase in the percentage of clay particles in the MC and all intercropping treatment soils (*P* < 0.05), with Macs having the smallest percentage of soil particle size, while the percentages of clay, silt, and Vfs in the soils were roughly similar.

### 3.4 Plant–soil relationship

**Figure 7:**
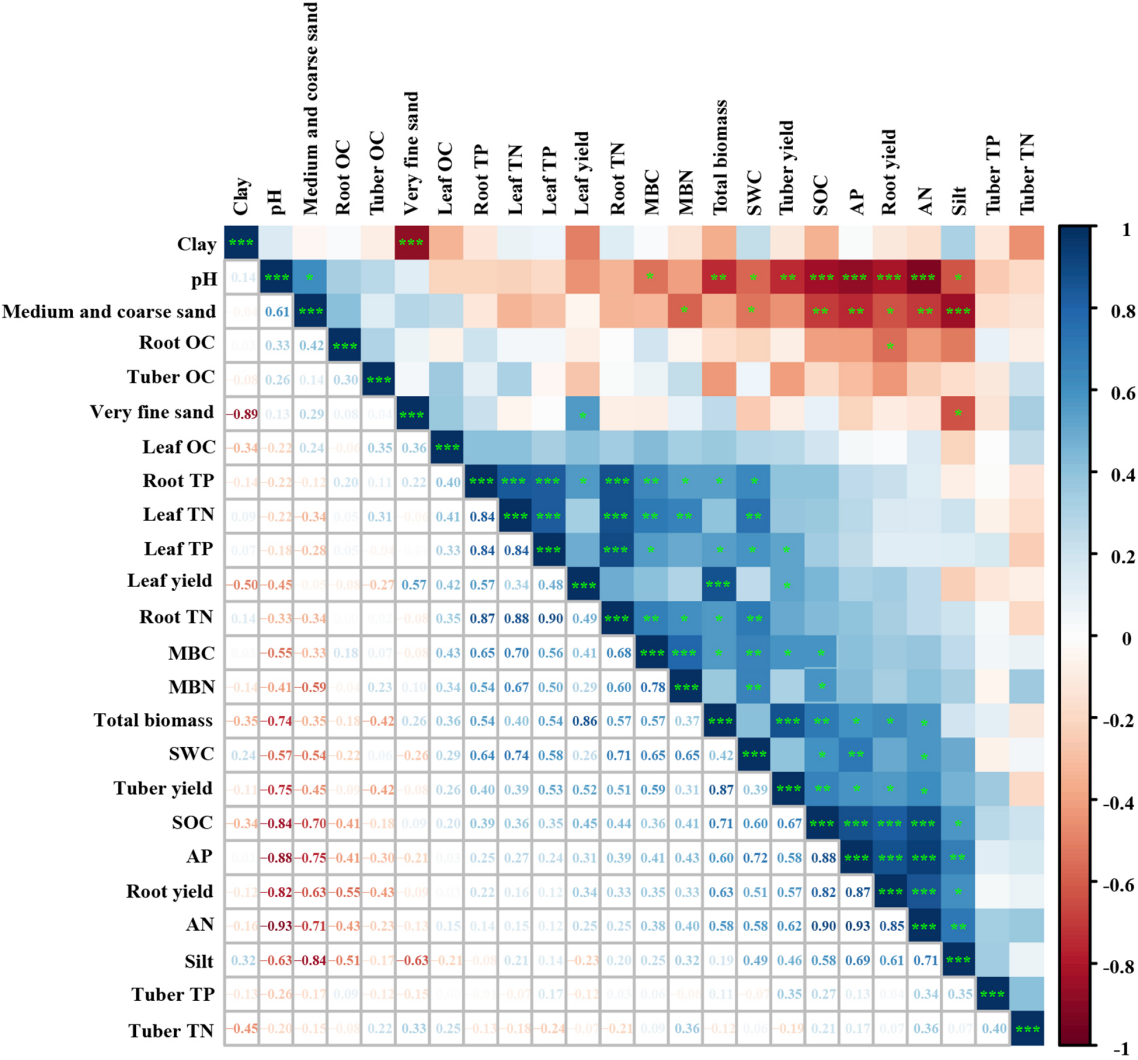
Relationships between plant and soil. *P < 0.05, **P < 0.01, ***P < 0.001.

The SOC, AN, and AP were significantly and positively correlated with the total biomass of *C. esculentus* (*P* < 0.05, **Figure 7**). The MBC and MBN were significantly and positively correlated with the total biomass, TN and TP in leaves, TN and TP in roots, and SOC in *C. esculentus* (*P* < 0.05). Similarly, the SWC was significantly and positively correlated with the TN and TP in leaves and roots, soil nutrients, the MBC, and MBN (*P* < 0.05). At the same time, significant negative correlations were observed between pH and total biomass and between soil nutrients and SWC (*P* < 0.05). In terms of soil particle size, a significant negative correlation was found between clay and Vfs, Macs, and the SOC and between AN and AP (*P* < 0.05).

## 4 Discussion

### 4.1 Plant growth response to different intercropping treatments

Intercropping, especially with legumes, confers substantial advantages in crop yield compared with monocropping (Qu et al., 2022; Lan et al., 2023; Parvin et al., 2023). This finding aligns with that of the present study, demonstrating higher *C. esculentus* biomass in intercropping treatments than in MC. This increase can be attributed to the legumes’ ability to achieve biological N fixation, thus alleviating N limitations in crops and fostering enhanced plant growth and development (Zhao et al., 2022). Moreover, intercropping optimizes soil nutrient management and fortifies crop nutrient supply, ultimately augmenting plant biomass (Zhang et al., 2016; Eusun et al., 2022; Lu et al., 2024). Conventionally, a higher proportion of the main crop correlates with increased yield (Tehulie and Nigatie, 2023). However, the present study revealed no significant differences in *C. esculentus*, alfalfa, and soybean yields among treatments with varying intercropping proportions. This finding suggests that the competitive effect of *C. esculentus* may outweigh the promoting effect of legumes in high-proportion cropping practices.

Beyond yield enhancements, intercropping with legumes increases the essential nutrient content in plants, particularly N and P (Schwerdtner and Spohn, 2022; Zhao et al., 2022). Furthermore, our results showed that the TN and TP contents of *C. esculentus* leaves and roots were significantly increased compared with the MC treatment. This study thus presents findings that are consistent with previous results—for example, the research of Li et al. (2022) on corn–soybean intercropping demonstrated significantly higher TN and TP contents in corn leaves compared with MC or corn–millet intercropping. Similarly, Li et al. (2010) observed a 9%–30% increase in maize’s N uptake in intercropping scenarios compared with MC. This increased N content in *C. esculentus* may be attributed to the process of legumes transferring nutrients to non-legumes (Chu et al., 2004; Rau et al., 2023). At the same time, the improvement in P nutrition in *C. esculentus* through intercropping may be attributed to P uptake released during legume root residue decomposition (Li et al., 2003). Furthermore, the enhanced N uptake promotes root growth and spatial distribution while delaying root senescence at the same time. Thus, the resulting boost in root uptake capacity contributes to the increased N content in *C. esculentus* (Zheng et al., 2021). These factors collectively underscore the multifaceted benefits of intercropping strategies involving legumes.

### 4.2 Response of soil properties to different intercropping treatments

Intercropping increases soil nutrients, thereby boosting crop yield and nutrient uptake capacity (Liu et al., 2014; Chamkhi et al., 2022). In particular, Lu et al. (2023) demonstrated that intercropping maize and soya bean increased AN and AP in inter-root soils. Similarly, Cuartero et al. (2022) concluded that the intercropping of melon with cowpea significantly increased the TC, TN, and AP levels in the soil compared with monocropping. Consistent with previous studies, our study revealed that intercropping *C. esculentus* with legumes significantly increased SOC, AN, and AP, thus enriching soil nutrients. This enhancement may be attributed to the increased populations of bacteria involved in soil nutrient cycling, such as rhizobia, phosphate-solubilizing, and potassium-solubilizing bacteria (Duchene et al., 2017; Zhang et al., 2018).

Moreover, distinct differences were observed among various intercropping practices (Qu et al., 2023), with the SOC, AN, and AP contents being significantly higher in *C. esculentus* and soybean intercropping treatments than in *C. esculentus* and alfalfa intercropping treatments. This finding may be attributed to the fact that the intercropping of *C. esculentus* with soybean increases the relative abundance of proteobacteria, which may potentially enhance N_2_ fixation (Rahav et al., 2016; Li et al., 2018; Huang et al., 2022; Jamali et al., 2023) and promote soil fertility. Among the intercropping of *C. esculentus* and soybean, soil nutrients were again significantly higher in the 4:4 intercropping treatment than in the 8:4 treatment. This is because the reduced competition intensity stemming from the low-proportion planting density of *C. esculentus* (Edmeades and Daynard, 1979; Tetio-Kagho and Gardner, 1988) decreases the competition for soil nutrient resources (Hara, 1986; Weiner, 1990; Schwinn and Weiner, 1998; Hara, 2005). In addition, the competition between *C. esculentus* and soybean may be lower than that with alfalfa, thus appearing to be significantly highest in the SOC, AN, and AP levels under the 4:4CG treatment.

The results of our study also showed a significant decrease in the pH level and a significant increase in the SWC of soil when *C. esculentus* was intercropped with legumes. These results are supported by previous studies, such as that of Wang et al. (2023), who observed a considerable decrease in soil pH after intercropping tea with legumes compared with monocropping. Shen et al. (2023) reported increased SWC when intercropping maize and soybean. These findings can be explained by the fact that intercropping with legumes can increase N fixation and decrease pH by increasing N accumulation (Chen et al., 2023; Liu et al., 2023). In addition, acidic metabolites are produced by intercropping plants with alfalfa and soybeans, resulting in a decrease in pH. These acids act as main osmotic pressure regulators, contributing to the increased SWC in intercropping (Rezig et al., 2013). Our results also showed that intercropping with legumes increases soil SWC, which is supported by previous studies indicating that intercropping systems can significantly improve soil physicohydraulic properties and optimize soil structure (Chen et al., 2019; Shen et al., 2023). This finding suggests that the intercropping of oilseed bean with soybean significantly improves adaptation to drought and provides a basis for promoting soybean cultivation in arid regions. The current study also revealed that, unlike the grain size of the IS, monocropping and intercropping both increased the clay percentage and reduced the percentage of Macs compared with the original soil’s grain size. However, no significant difference was observed in soil particle size between the MC and intercropping treatments, possibly due to the study’s short duration. Thus, additional experiments with extended durations must be conducted to further delve into this aspect.

Intercropping with legumes notably increased the concentrations of microbial C and N in the soil. This finding aligns with that of Qu et al. (2022), who revealed significant increases in MBC and MBN under intercropping, directly proving the notion that enhanced stimulation of the microbial community can be achieved through intercropping. This enhancement may stem from the strong correlation observed between total microbial biomass and soil C concentration (Goyal et al., 1993; Elfstrand et al., 2007). Notably, 4:4CG intercropping exhibited the highest MBC and MBN contents, which are likely due to the increased soil nutrient concentration that encourages microbial colonization and accumulation, subsequently boosting soil microbial activity (Wu et al., 2016). These alterations in soil properties and the subsequent increase in soil microbial population may have exerted a positive feedback on plant growth and productivity (Hauggaard-Nielsen and Jensen, 2005; Adomako et al., 2022). Therefore, further studies are needed to assess the feedback effects with microorganisms under *C. esculentus* intercropping.

## 5 Conclusions

The evaluation of *C. esculentus* yield, nutrient content, and soil properties within a *C. esculentus* and legume intercropping system encompassed various intercropping methods and ratios. The study results showed that intercropping significantly increased the yield and nutrients of *C. esculentus* compared with monocropping, although there were no significant differences between the intercropping treatments. This finding suggests that increasing the proportion of *C. esculentus* in intercropping does not increase the yield and nutrient content of the former. Furthermore, intercropping with legumes was not effective in counteracting the competitive effects of high proportions of *C. esculentus*. Meanwhile compared with monocropping, intercropping had significant positive effects on soil nutrients, with the 4:4CG treatment being the most effective. Therefore, the use of 4:4CG intercropping treatment is important to improve yield, plant nutrients, and soil quality, especially in arid and nutrient-deficient lands, such as Xinjiang. In addition, designing long-term field observations to understand the interaction mechanism of intercropping between oilseed rape and legumes is an important direction for future research.

## Data availability statement

The original contributions presented in the study are included in the article/supplementary material. Further inquiries can be directed to the corresponding authors.

## Author contributions

XS: Writing – original draft, Writing – review & editing. YL: Writing – review & editing. XL: Writing – review & editing. LL: Writing – review & editing.
